# Are floating toes associated with lifestyle in children? A cross-sectional study

**DOI:** 10.1186/s13047-023-00685-1

**Published:** 2023-12-13

**Authors:** Hideaki Nagamoto, Takumi Okunuki, Shimpei Takahashi, Kazuki Wakamiya, Zijian Liu, Toshihiro Maemichi, Hirofumi Katsutani, Yoshiyasu Yamada, Hiroyuki Takahashi, Hirofumi Tanaka, Toshimi Aizawa, Tsukasa Kumai

**Affiliations:** 1 Graduate School of Sport Sciences, Waseda University, 2-579-15 Mikajima, Tokorozawa, Saitama 359-1192 Japan; 2 Department of Orthopaedic Surgery, Tohoku University, Sendai, Miyagi Japan; 3 Department of Rehabilitation, Faculty of Medical Science and Welfare, Tohoku Bunka Gakuen University, Sendai, Miyagi Japan; 4 Specified Non-Profit Organization, Network for Sports Medicine and Science, Sendai, Miyagi Japan; 5 Japan Society for the Promotion of Sciences, Tokyo, Japan; 6 Institute of Life Innovation Studies, Toyo University, Tokyo, Japan; 7 Department of Physical Medicine and Rehabilitation, Tohoku University Graduate School, Sendai, Miyagi Japan; 8 Department of Orthopaedic Surgery, Kesen-Numa City Hospital, Kesen-Numa, Miyagi, Japan; 9 Faculty of Sport Sciences, Waseda University, Tokorozawa, Saitama Japan

**Keywords:** Floating toes, Lifestyle, Ankle dorsiflexion, Children

## Abstract

**Background:**

Floating toes are a condition and deformity in which some of the toes are afloat. Many functional impairments in floating toes have been previously studied lately and several factors related to floating toes have also been reported. However, no reports have considered the relationship between lifestyle and floating toes among children. The purpose of this study was to reveal the prevalence of floating toes among school children and reveal its relationship with lifestyle.

**Methods:**

In total, 138 young male baseball players were recruited. Lifestyle was evaluated by using a questionnaire and chosen whether the main lifestyle was Japanese or Western, if the bedding was futons or beds, and if the toilet was Japanese style (a squat toilet) or Western style. Floating toes were defined as toes that were not in contact with the mat. Ankle dorsiflexion in the knee-flexed and knee-extended positions was measured in a weight-bearing position. The relationship between the floating toes and lifestyles, and the comparison of ankle dorsiflexion range of motion between the lifestyles were statistically analyzed.

**Results:**

Players living in a Western style showed a significantly higher prevalence of floating toes on both feet compared with the players living in a Japanese style (throwing side; 39% vs. 19%, *p* = 0.04, and non-throwing side; 43% vs. 19%, *p* = 0.01). Players living in a Western style with beds showed a significantly smaller range of motion on both sides of ankle dorsiflexion in the knee-flexed position compared with those who were not (throwing side; 37.2 ± 5.7° vs. 39.0 ± 6.6°, *p* = 0.04, and non-throwing side; 36.8 ± 5.8° vs. 38.6 ± 6.1°, *p* = 0.04).

**Conclusion:**

Children mainly living in a Western lifestyle showed a significantly higher prevalence of floating toes on both feet compared to those mainly living in a Japanese lifestyle. The prevalence of floating toes may be related to lifestyles among children.

**Trial registration:**

The study was approved by the institutional review board of the Waseda University Graduate School of Sport Sciences (IRB number 2021–185).

## Introduction

Floating toes are a condition and deformity of the toes in which all the toes do not contact the ground while standing [1–7]. Many functional impairments in floating toes have been previously reported. A decrease in toe-grip strength caused by weakened toe flexors [1, 3], the ability to forwardly shift the center of gravity [1], a reduction in mechanoreceptor function [1], and a smaller base of support due to reduction in toe contact area are representative impairments [4]. Functional impairments are not limited to static functions but also dynamic functions, such as a decrease in dynamic balance, step length, and walking speed [4]. It also affects other parts of the body, increasing mechanical stresses on the knee and the lower back [4]. A possible relationship between floating toes and throwing injuries in baseball players has recently been reported [7]. Studies regarding floating toes have gained attention in recent years, as the prevalence of floating toes has been reported to increase among children in Japan [2, 8, 9].

Several factors related to floating toes have been reported, including physique, physical fitness, balance, shoes, heel load, exercise quantity, and frequency of toe use [2, 8, 9]. Also, genetic factors, inflammatory conditions, neuromuscular or metabolic disease, and post-surgical complications of Weil osteotomy, which causes the toes to afloat by an imbalance between the intrinsic and extrinsic muscles [10–12]. Recent studies have revealed that foot morphology, child’s age in months, height, and weight were factors that showed strong correlations with floating toes [2]. Besides physical factors, lifestyle factors like walking barefoot [13], and wearing socks [14] have also been studied. The reason that these kinds of factors has been reported is that many people in Japan live a traditional lifestyle: Japanese style, where you take off your shoes when you enter the house, sit directly on the tatami mat (Japanese straw floor coverings) which requires floor-sitting, squatting, and kneeling, and use futons to sleep rather than sit on a chair or couch while wearing shoes and sleeping on the bed in a Western style [15–17]. Previous literature has reported that Japanese lifestyle requires to squat deeply and frequently [18–20]. In addition, to squat deeply, ankle dorsiflexion is required [21]. Therefore, it can be assumed that living in Japanese lifestyle requires adequate ankle dorsiflexion angle and that those could be related to each other. However, to the best of our knowledge, there have been no reports on lifestyle and floating toes, and relationship between ankle dorsiflexion angle and lifestyles among children. Since many negative aspects related to floating toes have been elucidated and the prevalence of floating toes has been increasing lately [2, 8, 9], revealing the factors related to floating toes is necessary. Therefore, this study aims to reveal the prevalence of floating toes among school children and reveal its relationship with lifestyle.

## Methods

**Fig. 1:**
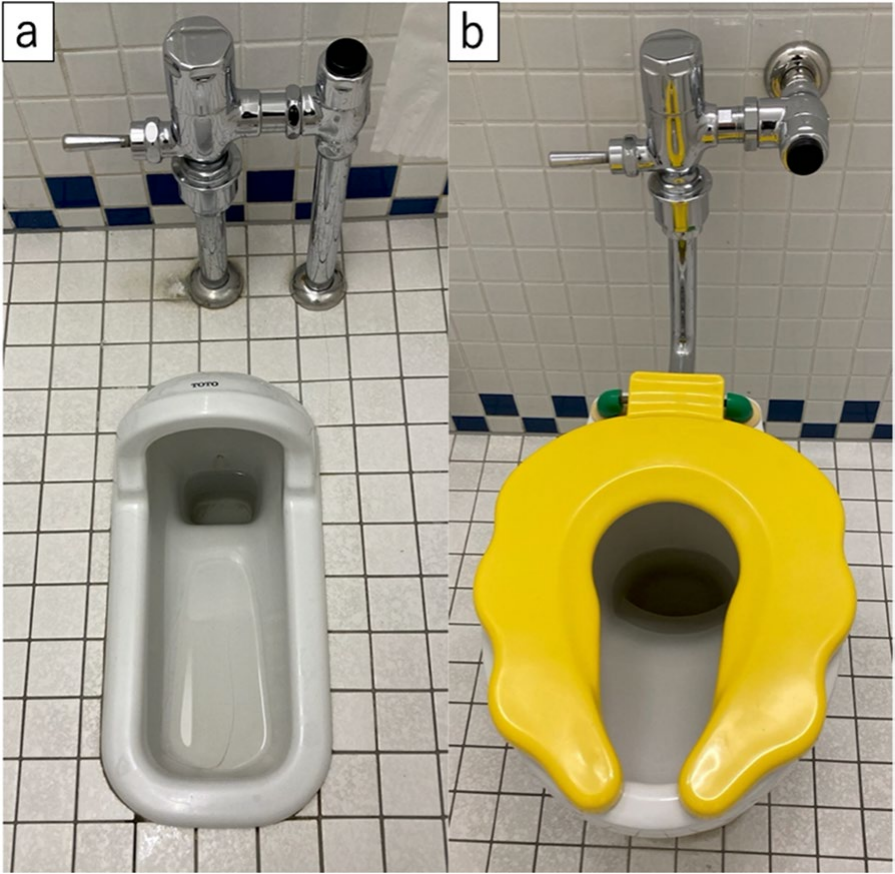
Images of toilet. (**a**) Japanese style toilet (a squat toilet); (**b**) Western style toilet

**Fig. 2:**
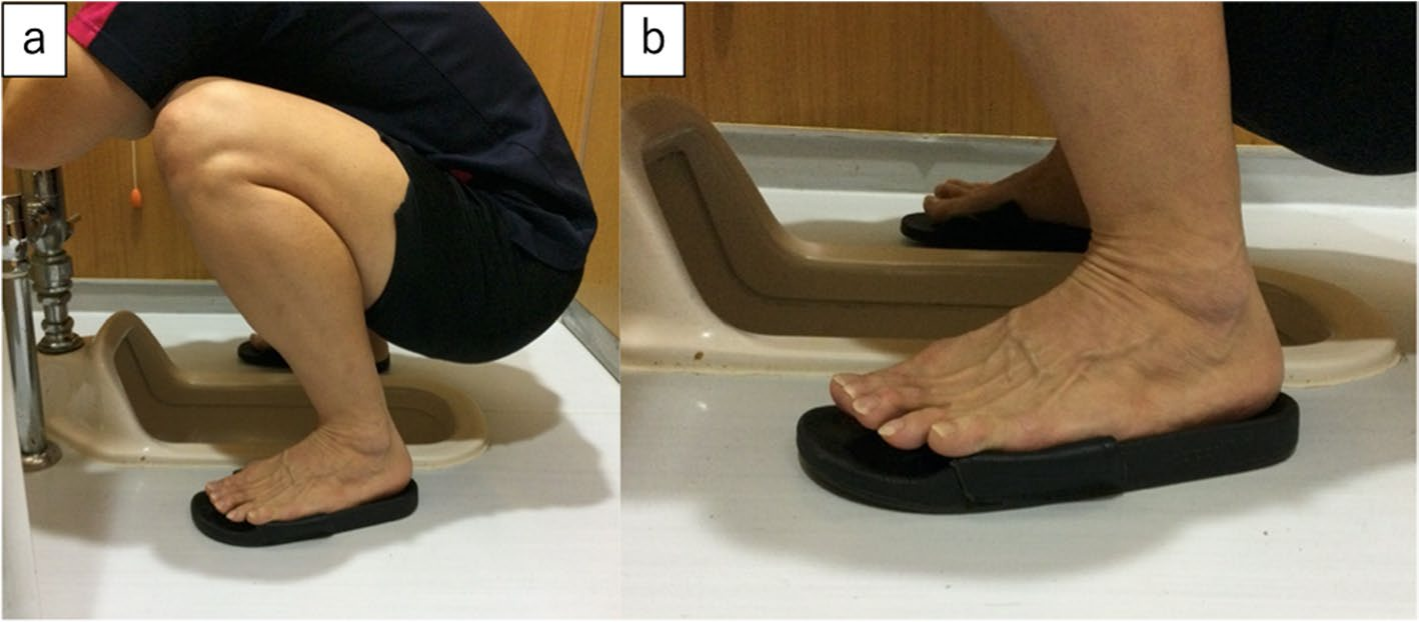
Images of positioning during the use of Japanese style toilet (a squat toilet); (**a**) Overview of the positioning; (**b**) Toes may float if the ankle dorsiflexion is restricted

This is a cross-sectional study. To gather information from a relatively uniform population, this study was conducted using data from the preseason clinical examinations of the young baseball players. The study was approved by the Ethical Review Committee on Research Involving Human Subjects of the senior author’s institution (IRB number 2021–185). Parents or the guardians, along with the players, provided written informed consent after the purpose, methods, and ethical considerations of the study were explained. A total of 139 young male baseball players were recruited in the study. Players were excluded if they had undergone surgery or sustained an injury to the foot in the past, apparent anatomical deformity involving toes, inflammatory conditions around the foot and toes, history of metabolic or neuromuscular disease, or if the questionnaire was incomplete. A questionnaire was provided in advance to ask about the lifestyle at home. The lifestyle was selected according to the previous literature regarding Japanese lifestyle [22]. It consisted of three questions: whether the main lifestyle was Japanese or Western, if the bedding was futons or beds, and if the toilet was Japanese style (a squat toilet) or Western style (Fig. 1). Specific positioning of using the Japanese style toilet is shown in Fig. 2. If the ankle dorsiflexion is restricted, toes may float, shown in the figure (Fig. 2-b).

**Fig. 3:**
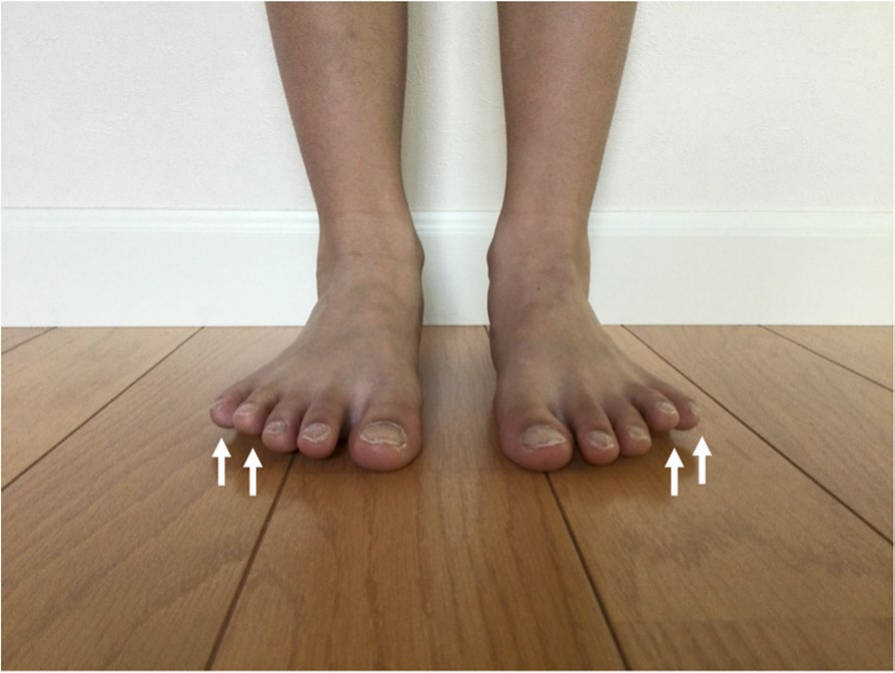
Typical figure of floating toes. White arrows indicate floated toes

Floating toes were evaluated while players stood upright on a firm mat. After instructing the players to keep their feet in shoulder-width apart, they were instructed to stare at the marker, which was 2 m in front of them at eye level. Floating toes were defined as toes that were not in contact with the mat (Fig. 3). This definition and the method are in accordance with the previous reports [1, 5, 7]. As a part of preseason clinical examination, the ankle dorsiflexion range of motion was measured. Ankles were measured in the weight-bearing lunge position using a standard goniometer. The weight-bearing lunge position was instructed with the players facing a wall and both of the heels in contact with the ground at all times. The knee was kept in line with the line of the second metatarsal, and the hallux was located 10 cm away from the wall while the players maintained their balance by placing their fingers on the wall. The players were then instructed to further lunge forward toward the wall, keeping the direction of the knee aligned with the second metatarsal, until their knee made contact with the wall. After this forward lunge position was confirmed, players were instructed to move the ipsilateral foot away from the wall 1 cm at a time until they were unable to maintain contact of the knee with the wall without lifting their heel off the floor. Ankle dorsiflexion in the knee-flexed position (DFKF) was measured in the final lunge position. After measuring the DFKF, the knee on the contralateral side was also kept in line with the second metatarsal line and extended with the heel in contact with the floor. The players were instructed to lean forward until maximal stretching of the posterior leg was felt. The contralateral ankle was also measured as the ankle dorsiflexion in the knee-extended position (DFKE). For both angles, the stable arm of the goniometer was parallel to the fifth metatarsal while the mobile arm was parallel to the shaft of the fibula, and the angle between the arms was recorded [23].

The prevalence of floating toes and the percentage of each lifestyle were calculated. A chi-square test was performed to determine the relationship between the floating toes and lifestyles. Comparisons of DFKF and DFKE between the lifestyles were analyzed using a Student’s t-test. Then, DFKF and DFKE were compared between regrouped players: players living in a Western style with beds and those who were not. JMP Pro 15 (SAS Institute, Cary, NC, USA) was used for statistical analysis. The significance level was set at *p* < 0.05. Power analysis was conducted before the examination. Power of 0.8 at a significance level of 0.05 was set and the required sample size was 128.

## Results

**Fig. 4:**
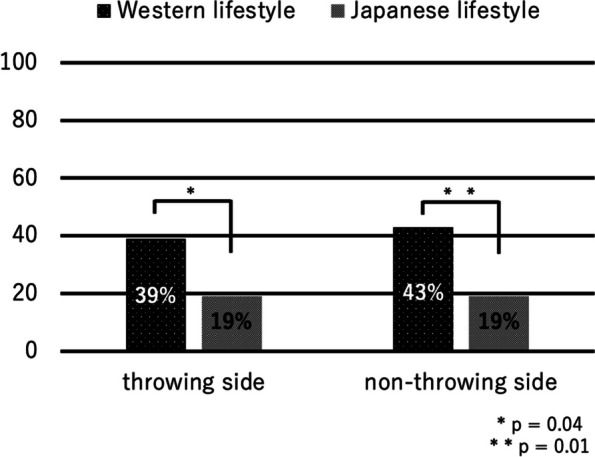
Comparison of prevalence of floating toes between lifestyles

**Fig. 5:**
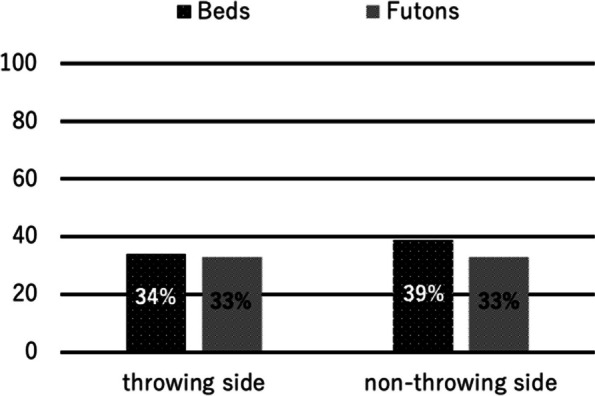
Comparison of prevalence of floating toes between bedding

Among 139 male young baseball players, 138 players met the inclusion criteria and were enrolled in the statistical analysis. Their mean age was 11.2 ± 0.7 years (range, 10–12 years). Overall mean height and weight was 149.1 ± 7.9 cm (range, 130–170 cm), and 41.8 ± 8.3 kg (range, 25–65 kg), respectively. The prevalence of floating toes was 33% (*n* = 46) on the throwing side and 36% (*n* = 50) on the non-throwing side. Comparison of height and weight between those with floating toes and without floating toes showed no significant difference. Seventy-three percent of the players (*n* = 101) answered that their main lifestyle was Western, and 57% (*n* = 79) answered that their bedding was a bed. For the toilet, except for three players, all the players (98%) answered that their toilet was a Western style. Players living in a Western style showed a significantly higher prevalence of floating toes on both feet compared with players living in a Japanese style. On the throwing side, the prevalence of floating toes among Western-style living players was 39% (*n* = 39), whereas that among Japanese-style living players was 19% (*n* = 7 players; *p* = 0.04). On the non-throwing side, the prevalence of floating toes among Western-style living players was 43% (*n* = 43), whereas that among Japanese-style living players was 19% (*n* = 7; *p* = 0.01) (Fig. 4). Comparison between bed and futon did not show any significance in prevalence of floating toes in both throwing and non-throwing sides (Fig. 5). As for the toilets, since there were only 3 players who answered that their toilets were Japanese style, statistical analysis could not be performed.

The average range of motion of the DFKF on the throwing and non-throwing sides was 38.1 ± 6.2° (95% confidence intervals [CI], 37.1–39.2°) and 37.8 ± 6.0° (95% CI, 36.8–38.8°), respectively. The average range of motion of DFKE on the throwing and non-throwing sides was 28.6 ± 6.1° (95% CI, 27.6–29.7°) and 28.5 ± 5.2° (95% CI, 27.6–29.4°), respectively. A comparison of the range of motion between the sides in both conditions was not statistically significant. The DFKF and DFKE data are summarized in Table 1. Players living in a Western style with beds (n = 65) showed a significantly smaller range of motion on both sides of DFKF (37.2 ± 5.7°; 95% CI, 35.8–38.6° and 36.8 ± 5.8°; 95% CI, 35.4–38.3° in the throwing and non-throwing sides, respectively) compared with those who were not (*n* = 73) (39.0 ± 6.6°; 95% CI, 37.4–40.5° and 38.6 ± 6.1°; 95% CI, 37.2–40.1° in the throwing and non-throwing sides, respectively; *p* = 0.04 for both sides). However, there was no significant difference in range of motion on both sides of the DFKE or in the other comparisons.

**Table 1:** Results of ankle dorsiflexion angle. *T-DFKE* Throwing side ankle dorsiflexion in knee-extended position, *NT-DFKE* Non-throwing side ankle dorsiflexion in knee-extended position, *T-DFKF* Throwing side ankle dorsiflexion in knee-flexed position, *NT-DFKF* Non-throwing side ankle dorsiflexion in knee-flexed position. *p = 0.04, **p = 0.04

Grouping categories	T-DFKE	NT-DFKE	T-DFKF	NT-DFKF
With floating toes on the throwing side (*n* = 46)	29.2 ± 5.1° (95% CI, 27.7–30.8°)	28.5 ± 4.5° (95% CI, 27.2–29.8°)	39.1 ± 6.1° (95% CI, 37.3–40.9°)	38.7 ± 5.4° (95% CI, 37.1–40.3°)
Without floating toes on the throwing side (*n* = 92)	28.4 ± 6.5° (95% CI, 27.1–29.7°)	28.5 ± 5.5° (95% CI, 27.3–29.6°)	37.6 ± 6.1° (95% CI, 36.6–38.9°)	37.3 ± 6.3° (95% CI, 36.0–38.6°)
With floating toes on the non-throwing side (*n* = 50)	29.2 ± 5.3° (95% CI, 27.7–30.7°)	28.3 ± 4.1° (95% CI, 27.1–29.5°)	38.6 ± 5.3° (95% CI, 36.8–40.4°)	38.1 ± 5.5° (95% CI, 36.5–39.7°)
Without floating toes on the non-throwing side (*n* = 88)	28.4 ± 6.5° (95% CI, 27.0–29.7°)	28.6 ± 5.7° (95% CI, 27.4–29.8°)	37.9 ± 6.2° (95% CI, 36.6–39.1°)	37.6 ± 6.3° (95% CI, 36.3–39.0°)
Japanese lifestyle (*n* = 37)	28.4 ± 6.0° (95% CI, 26.4–30.3°)	28.4 ± 5.1° (95% CI, 26.7–30.0°)	38.4 ± 6.4° (95% CI, 36.4–40.4°)	38.5 ± 5.3° (95% CI, 36.5–40.4°)
Western lifestyle (*n* = 101)	28.8 ± 6.1° (95% CI, 27.5–29.9°)	28.5 ± 5.2° (95% CI, 27.5–29.5°)	38.0 ± 6.2° (95% CI, 36.8–39.2°)	37.6 ± 6.3° (95% CI, 36.4–38.8°)
Futons (*n* = 59)	29.6 ± 7.0° (95% CI, 27.7–31.4°)	29.0 ± 5.8° (95% CI, 27.5–30.5°)	39.1 ± 6.8° (95% CI, 37.3–40.9°)	38.7 ± 6.5° (95% CI, 37.0–40.4°)
Beds (*n* = 79)	28.0 ± 5.3° (95% CI, 26.8–29.2°)	28.1 ± 4.7° (95% CI, 27.1–29.2°)	37.4 ± 5.7° (95% CI, 36.2–38.7°)	37.2 ± 5.6° (95% CI, 35.9–38.4°)
Japanese toilet (*n *= 3)	30.0 ± 5.0° (95% CI, 17.6–42.4°)	33.3 ± 7.6° (95% CI, 14.4–52.3°)	40.0 ± 8.7° (95% CI, 18.5–61.5°)	40.0 ± 5.0° (95% CI, 27.6–52.4°)
Western toilet (*n* = 135)	28.6 ± 6.1° (95% CI, 27.6–29.7°)	28.4 ± 5.1° (95% CI, 27.5–29.3°)	38.1 ± 6.2° (95% CI, 37.0–39.1°)	37.8 ± 6.1° (95% CI, 36.7–38.8°)
Western lifestyle and beds (*n* = 65)	28.3 ± 5.5° (95% CI, 26.9–29.7°)	28.2 ± 4.9° (95% CI, 27.0–29.4°)	*37.2 ± 5.7° (95% CI, 35.8–38.6°)	**36.8 ± 5.8° (95% CI, 35.4–38.3°)
Neither a Western lifestyle nor beds (*n* = 73)	28.9 ± 6.6° (95% CI, 27.4–30.5°)	28.7 ± 5.6° (95% CI, 27.4–29.9°)	*39.0 ± 6.6° (95% CI, 37.4–40.5°)	**38.6 ± 6.1° (95% CI, 37.2–40.1°)

## Discussion

Our findings revealed that children who primarily lived in Western lifestyles had a significantly higher prevalence of floating toes on both feet than those who primarily lived in Japanese lifestyles. Furthermore, the ankle dorsiflexion range of motion of both sides in the knee-flexed position was significantly smaller in players who lived in a Western style with beds compared with those who did not. One study investigated about the relationship between the ability to squat deeply and lifestyle among elementary school children [16]. The results revealed that children who were able to squat deeply (squat without leaving their heels off the floor) were significantly more common among those sleeping on futons compared to those sleeping on beds. Furthermore, children sleeping in futons showed higher rates of living in a Japanese style than those using chairs. These results may suggest that children living in a Japanese style and sleeping on futons may have to squat more frequently and deeply compared with those living in a Western style and sleeping on beds. It has been previously reported that those sleeping on a futon or sitting on a tatami are required to frequently squat [18]. Therefore, those living mainly in a Japanese style and sleeping on futons may show greater ankle dorsiflexion compared with those living mainly in a Western style and sleeping on beds. This could be emphasized by our results of ankle dorsiflexion range of motion; the combination of a Western style and bed showed significant results rather than independent analysis by a Western style or beds. Previous literature has revealed that those with restricted ankle dorsiflexion cannot squat deeply and that also showed posterior displacement in center of pressure [19]. Another study has reported that those with floating toes showed significant posterior displacement in center of pressure compared to those without it [24]. Matsuda et al. have revealed that posterior inclination induces increase in muscle activity of the tibialis anterior muscle [25]. As the tibialis anterior is an extrinsic muscle, over activation of the tibialis anterior due to posterior displacement can lead to imbalance between the intrinsic and extrinsic muscles, which can result in the occurrence of floating toes. Indeed, past report has revealed that those with floating toes shows decreased intrinsic muscles, especially the toe flexors and the toe-grip strength [13]. In addition, a recent study revealed that young baseball players who were unable to squat deeply showed significantly higher rates of combining impaired foot function compared with those who were able to squat deeply [26]. Along with these previous reports and our results strongly suggests that children who cannot squat deeply, or having restricted ankle dorsiflexion angle in knee-flexed position tended to have functional impairment of the toes for having floating toes. However, as the previous studies and our results only consisted of Japanese participants, applying the lifestyle or results to other ethnicities or cultures should be interpreted with discretion.

The range of motion of the ankle dorsiflexion is commonly affected by gastrocnemius tightness [27, 28]. However, when the ankle is dorsiflexed in the knee-flexed position, the effect of the gastrocnemius diminishes because it is a biarticular muscle, bridging the knee and the ankle. When the ankle dorsiflexion in the knee-flexed position is restricted, other structures around the ankle besides the gastrocnemius are responsible, including osseous structures, ligaments, or muscular structures (i.e., the soleus, and plantar fascia) [27, 28]. To squat deeply, adequate joint range, not only in ankle dorsiflexion but also in the knee flexion, and hip flexion, is required [29]. In our study, players living in a Western style with beds showed significantly decreased ankle DFKF. Therefore, these players may have structural stiffness or lack of flexibility surrounding the ankle joint. However, as there was no significant relationship between the prevalence of floating toes and the ankle dorsiflexion angle, other functional factors may have affected the ability to dorsiflex the ankle adequately or the occurrence of the floating toes. Future studies should reveal which factors are related to a decreased ankle dorsiflexion angle in the knee-flexed position among children and also other physical and functional factors related to floating toes.

The prevalence of floating toes among children (an average age of 10.2 years in males and 10.3 years in females) has been reported to be 40.3% [3] and 41.8% in adults (an average age of 22.8 years) [5]. The results of our study (33–36%) seem reasonable given previous reports. In contrast, Araki et al. [2] reported a significantly higher prevalence of 87% among children aged three to five years. This difference may be due to differences in age between the participants and developmental factors. In fact, they have suggested that floating toes are affected by motor development, environmental factors, or lifestyle factors. Our study suggests that environmental factors may play a role in the development of floating toes. Longitudinal studies should be performed on children in the future to reveal the factors related to the occurrence of floating toes further.

This study has several limitations that must be mentioned. The participants were limited to young male baseball players. Previous literatures have reported that the prevalence of floating toes differs between sex and that the prevalence in male was lower than female [30, 31], which is the main reason we only recruited only male participants. Therefore, applying the results to general young population in Japan and in girls requires careful discretion. The ankle dorsiflexion angle was measured by several therapists. As the study was conducted as part of the preseason clinical examination, the measurements had to be shared. Nevertheless, the measurement of ankle dorsiflexion has been reported to be highly reliable, regardless of the measurement methods [32, 33]. Therefore, it was assumed that the measurement method did not affect the results. Lifestyles may have differed in the past. However, due to its cross-sectional nature, lifestyle could only be investigated on the occasion of answering the questionnaire. Thus, length of the lifestyle should be considered in the future studies. The most important finding of this study was that the prevalence of floating toes may be related to the lifestyles of the children.

## Conclusions

Children mainly living in a Western lifestyle showed a significantly higher prevalence of floating toes on both feet compared to those mainly living in a Japanese lifestyle. The ankle dorsiflexion range of motion of both sides in the knee-flexed position was significantly smaller in children living in a Western style with beds compared with those who were not. The prevalence of floating toes may be related to lifestyles among children with restricted ankle dorsiflexion angle in knee-flexed position and further studies are required to reveal other relevant physical and functional factors.

## Data Availability

The datasets used and/or analyzed during this study are available from the corresponding author on reasonable request.
